# mSphere of Influence: Deterministic and Stochastic Processes Drive Microbial Evolution

**DOI:** 10.1128/msphere.00022-23

**Published:** 2023-02-07

**Authors:** Aleeza C. Gerstein

**Affiliations:** a Department of Microbiology, The University of Manitoba, Winnipeg, Manitoba, Canada; b Department of Statistics, The University of Manitoba, Winnipeg, Manitoba, Canada

**Keywords:** drift, evolution, experimental evolution, fitness, genetics, mutation, selection

## Abstract

Aleeza C. Gerstein works in the field of microbial evolutionary genetics in human fungal pathogens. In this mSphere of Influence article, she reflects on how the papers “Experimental tests of the roles of adaptation, chance, and history in evolution” by Travisano et al. (1995) and “Pervasive genetic hitchhiking and clonal interference in forty evolving yeast populations” by Lang et al. (2013) provided her with a framework for thinking about the competing stochastic and deterministic evolutionary forces that act on microbial populations at the genotypic and phenotypic levels to cause both parallelism and divergence under different scenarios.

## COMMENTARY

The first time I formally encountered evolutionary biology in my high school biology class, I was immediately hooked. Suddenly, I had language and a theoretical framework to help me to organize my understanding of the world around me. Mutations arise randomly (more or less) in genomes at some given rate (μ), and the acquisition of beneficial mutations enables populations to become better adapted to their abiotic and biotic environments. The rate of adaptation depends on the population size (*N*) and the effect size of the available mutations (*s*, scaled by the dominance parameter, *h*, in diploids).

Although I didn’t set out to be a microbiologist, when I got to graduate school I realized almost immediately that microbes provide ideal experimental systems to study the factors that influence evolutionary outcomes (with the bonus that there is hopefully never a storm, or drought, or cow wandering through your lab, influencing your experiments in unavoidable and undesirable ways). The first “Lenski-style” experimental evolution study was published in 1992 (1), documenting how bacterial populations can be evolved through batch culture in replicate from the same ancestor in identical environments to directly “replay life’s tape,” *à la* S. J. Gould and R.C. Lewontin (2). Since the ancestral populations can be preserved in the freezer and revived as required, ancestral and evolved genotypes can be directly compared and competed against each other to quantify similarities and differences in genotype, phenotype, and fitness among evolved replicates.

Many papers influenced my emerging microbially focused lens on evolutionary genetics, and chief among them was the 1995 paper by Travisano, Mongold, Bennett, and Lenski, “Experimental tests of the roles of adaptation, chance, and history in evolution” (3). Travisano et al. describe two sets of experiments that empirically compared evolved replicates, to understand the influences of history (attributable to differences in ancestral genotype), chance (observed as variation among evolved replicates due to the mutations that arose and their fixation probability), and adaptation (manifest as consistent phenotypic changes in evolved replicates). They built off the results of Lenski et al. (1), where 12 replicate lines were propagated for 2,000 generations at 37°C under glucose limitation; the evolved replicates had similar fitness under glucose limitation but were heterogeneous under maltose limitation. In the first experiment, one genotype from each evolved replicate became ancestral to three new populations, and all 36 lines were propagated for 1,000 additional generations under maltose limitation. In the second experiment, one genotype from each of the 36 evolved replicates was ancestral to an additional 24 populations, with six each propagated at 32°C, 37°C, or 42°C or with daily shifts from 32°C to 42°C for 2,000 generations. One genotype from each evolved population was then propagated at 20°C for an additional 1,000 generations. In both experiments, fitness (measured as competitive fitness between evolved and ancestral populations) was predominantly influenced by adaptation, as all replicates acquired similar evolved fitness. Increases in fitness were deterministic; beneficial mutations arose and were fixed rapidly enough to overcome historical differences in ancestral genotype and the potential influence of genetic drift. In contrast, evolved cell sizes were not correlated with fitness in either experiment and were much more variable (seemingly stochastic), indicating that phenotype was more influenced by chance and history.

In 2009, Barrick and Lenski published the first major paper on evolutionary genetics enabled by whole-genome sequencing, shedding light for the first time on the mutational dynamics that powered evolved phenotypic and fitness changes in one of the evolved Lenski lines (4). In 2013 when I was a new postdoctoral fellow, Lang et al. extended this achievement in “Pervasive genetic hitchhiking and clonal interference in forty evolving yeast populations” (5). They sequenced 40 haploid yeast populations evolved in rich medium for 1,000 generations at 12 time points, to 100-fold sequencing depth. This enabled them to ask big-picture evolutionary questions about constraint and parallelism similar to those of Travisano et al. (3) but with high resolution at the genetic level. They found that ~25% of the identified mutations eventually fixed among the evolved populations. Surprisingly, they also found that genetic hitchhiking involving many mutations (“mutational cohorts”) was highly pervasive among evolved lines. Correspondingly, the loss of beneficial mutations was also common, due to interference among competing cohorts. Looking across replicates, the overall mutational dynamics beautifully illustrated the balance between determinism and stochasticity. Deterministic adaptation due to “driver mutations” was evident as parallelism in the genetic basis of adaptation; 24 genes were mutated in three or more lines. However, stochasticity determined whether driver mutations were able to sweep through the population in which they arose. There was often interference between mutational cohorts, and multiple beneficial mutations (“co-drivers”) were sometimes required to arise in the same genetic background for fixation. To use the terminology of Travisano et al. (3), from the perspective of a single beneficial mutation that arises in an individual in an evolving population, adaptation caused the same gene targets to be beneficial across replicates but history (the genetic background in which a beneficial mutation arises) and chance (competing mutational cohorts that exist in the population) all combined to determine the eventual dynamics.

The patterns and processes in the two focal papers almost seem simplistic upon reflection years later. Yet isn’t this the history and beauty of science? When I wrote my Ph.D. proposal on the characterization of beneficial mutations in Saccharomyces cerevisiae, I aspiringly waved my hands about attempting to pinpoint the genomic region where they arose; 3 short years later, we had simply whole-genome sequenced 60 independently evolved lines and precisely identified the amino acids changes. As a more recent example, how rapidly we have taken for granted the ability to precisely alter a single base pair in even a non-model organism genome!

It need not be the case that adaptation will always overwhelm history and chance, and much of my recent (and future) work seeks to identify scenarios in which historical contingency and chance might indeed play much larger roles, particularly in the context of antimicrobial resistance and virulence. The results from my lab and others have demonstrated that divergent genetic backgrounds influence evolutionary trajectories in many experiments. We have recently been surprised to discover that the dominant phenotype under selection in the presence of a drug in many *in vitro* contexts seems to be drug tolerance rather than drug resistance. The fungal community is beginning to understand the effects and evolutionary stability of aneuploidies and structural variants, compared to “typical” single-nucleotide changes and small indels, and determine how different types of mutations interact over different timescales. It is not yet clear how the presence of multiple stressors (as is inherent in populations adapting to complex environments, such as within the human body) influences constraints on genotypic or phenotypic evolution and, as sequencing enables us for the first time to characterize and “see” some microbial species that are difficult to grow in the lab, we are just beginning to explore the interplay between abiotic and biotic pressures on adaptation in natural microbial populations. I credit the papers described here with illuminating the need to always consider stochastic and deterministic factors to untangle and understand the evolution of (microbial) life.
